# Effects of *RIM15* deletion on yeast adaptation to synthetic spruce hydrolysate

**DOI:** 10.1186/s12934-026-03096-6

**Published:** 2026-08-15

**Authors:** Nathália Vilela, Cecilia Trivellin, Luca T Pianale, Julie Brouchon, Julien Patrouix, Diana Ekman, Simon Trancart, Lisbeth Olsson

**Affiliations:** 1 Department of Life Sciences, Division of Industrial Biotechnology, Chalmers University of Technology, 412 96 Gothenburg, Sweden; 2 Altar–Lesaffre Group, Genopole Campus 1, Evry, France; 3 Department of Biochemistry and Biophysics, National Bioinformatics Infrastructure Sweden, Science for Life Laboratory, Stockholm University, Solna, Sweden

**Keywords:** Bioethanol, Adaptive laboratory evolution, Transcription factors, Genome sequencing, Turbidostat, Bioprocess

## Abstract

Microbial robustness (i.e., to keep the same performance in the face of several perturbations) is a desirable trait for industrial yeasts, particularly in second-generation bioethanol production, where inhibitory compounds in lignocellulosic hydrolysates impair microbial performance. In this study, we investigated the role of the stress-responsive kinase Rim15 in stress adaptation and robustness by subjecting *Saccharomyces cerevisiae* wild-type (WT) and rim15Δ strain to adaptive laboratory evolution (ALE) in synthetic spruce hydrolysate (SSH). This hydrolysate contains high concentrations of inhibitory compounds, such as acetic acid and 5-hydroxymethylfurfural. Crucially, ALE consisted of two steps, including a medium swap regime that enabled adaptation to 100% SSH. Although WT and rim15Δ strain followed distinct evolutionary trajectories during ALE, both strains improved growth performance and robustness on SSH. The ability of the rim15Δ strain to adapt to 100% SSH was associated with impaired daughter cell separation and multicellular clump formation. Genome sequencing identified a mutation in a morphogenetic gene (*ACE2*) in Evolved rim15Δ. This differed from mutations in key transcriptional regulators (e.g., *SSN8*, *SSN2*) identified in Evolved WT. Reverse engineering indicated that *ACE2* loss-of-function contributes to the multicellular clumping phenotype of the Evolved rim15Δ strain, while deletion mutants of *SSN2* and *SSN8* exhibited improved growth in SSH compared to the parental strain. Together, these findings indicate that yeast robustness can emerge through distinct adaptive strategies shaped by the genetic background, involving transcriptional rewiring or morphogenetic adaptation to lignocellulosic stress.

**Graphical Abstract:**

**Figure:**
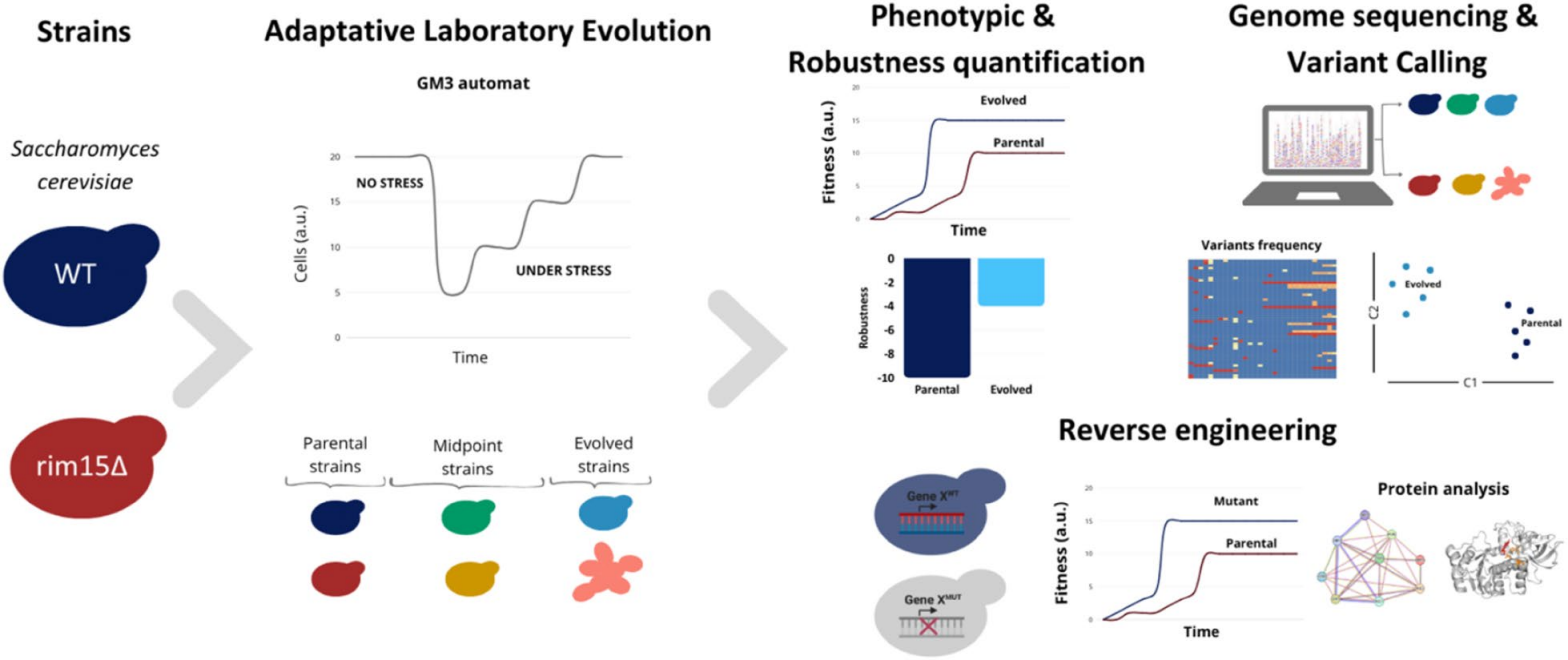

**Supplementary Information:**

The online version contains supplementary material available at 10.1186/s12934-026-03096-6.

## Introduction

Biorefineries convert renewable biomass into biofuels, energy, and value-added biochemicals using integrated bioprocessing. Economically viable and competitive biorefineries must apply different means to ensure fermentation efficiency. However, the multiple industrial stresses, including hydrolysate-derived inhibitory compounds and nutrient limitation, prevent strategies, such as increased strain tolerance or continuous control of fermentation parameters, from achieving the desired improvement. Environmental perturbations are often unpredictable or act synergistically, making them difficult to overcome. For example, in the production of second-generation biofuels, yeast encounters severe stress due to the presence of different growth inhibitors released during pre-treatment of lignocellulosic feedstocks [14]. A successful strategy would be to use robust strains, which maintain consistent performance under different perturbations [24]. To design robust strains, though, one must first understand which molecular traits are responsible for the desired robustness.

Yeast has evolved a sophisticated set of mechanisms that enable it to recognize and respond to stress caused by temperature fluctuations, nutrient deprivation, and the presence of inhibitory substances [7]. The stress response involves sensors, signaling pathways, and transcription factors (TFs) that activate genes related to carbon metabolism, transporters, proteases, and protective proteins [8]. The activated TFs include both generic stress-related TFs, exemplified by *MSN2* and *MSN4*, and stress-specific TFs, such as *HAA1*, *YAP1*, and *HSF1*, which are involved in weak acid stress, oxidative stress, and protein accumulation, respectively [31].

*MSN2/4* regulate the level of over 200 stress response genes by binding to the stress response element and mediating the expression of protein kinase A-dependent genes [30]. They also play vital roles in entering and exiting quiescence, regulating enzymes involved in carbohydrate metabolism, G1 arrest, trehalose, and glycogen accumulation, as well as starvation resistance, which are all essential when mitigating stress [17, 20, 27, 29]. The upstream activator, *RIM15*, regulates the *MSN2*/*4* genes [41]. Upon nutrient starvation or exposure to environmental stress, Rim15 phosphorylates Msn2/4, triggering their nuclear translocation and consequent downstream response [19].

Given this central regulatory role, *RIM15* is of interest in the context of industrial fermentation. Studies using modern sake *Saccharomyces cerevisiae* strains revealed a common loss-of-function mutation (5055insA) in *RIM15*, associated with increased fermentation rates due to impaired transition to the G0 phase, i.e., quiescence [39]. Similarly, *RIM15* deletion in the laboratory strain BY4743 enhances fermentation by compromising G0/G1 arrest [25, 39]. However, this benefit comes at a cost: loss of *RIM15* impairs stress response, decreases accumulation of storage carbohydrates, reduces thermotolerance and longevity, and increases the budding index [2]. While these issues may appear only late in sake fermentation, they are relevant in another industrial context, such as lignocellulosic biorefineries. Therefore, further investigation of this gene may reveal previously uncharacterized mechanisms underlying stress adaptation and microbial robustness, with potential relevance for industrial applications [40].

Microbial robustness has been defined as the ability to maintain consistent performance across different perturbation conditions, differing conceptually from stress tolerance, which is typically associated with growth under a single stress condition [24]. Robustness quantification is based on the data dispersion of biological functions across multiple perturbations, with a theoretical value of 0 indicating maximum robustness [36]. This framework has previously been applied for mutant library screening and strain characterization under industrially relevant perturbation spaces [35, 37].

In the present study, *RIM15* was deleted in the *S. cerevisiae* laboratory strain CEN.PK113-7D to determine how the removal of this stress regulator alters evolutionary trajectories under lignocellulosic stress. Adaptive laboratory evolution (ALE) has emerged as an excellent technique for improving yeast adaptability to more complex environments [23]. Here, we used a dual-chamber cultivation system for a two-step ALE experiment, whereby a medium swap compelled adaptation in 100% synthetic hydrolysate containing various inhibitory compounds. This evolution regime forced cells to adapt to stress conditions triggered by toxic liquid pulses, but also to rapid growth in nutrient-rich medium [22]. Subsequently, strain performance and robustness were analyzed, and whole-genome sequencing was performed to identify genetic variants in the evolved strains relative to the parental strain. This experimental framework provides insights into how genetic background influences adaptive trajectories in lignocellulosic stress, offering valuable guidance for future engineering of industrial yeast strains.

## Results and discussion

*S. cerevisiae* CEN.PK113-7D and CEN.PK113-7D rim15Δ were evolved in synthetic spruce hydrolysate (SSH) to assess how distinct genetic backgrounds shape adaptation to lignocellulosic stress. Adaptation to 100% SSH was first monitored through specific growth rate measurements, then performance was evaluated across multiple perturbations and robustness was quantified. Finally, the genetic basis of adaptation was identified and validated by whole-genome sequencing and reverse engineering.

### Stress adaptation to SSH occurs independently of RIM15

ALE was performed in a GM3 automat (Genemat), a continuous-culture device with automated control of environmental conditions. It featured a unique twin growth chamber designed to actively counter-select surface-attached or biofilm-forming cells, ensuring that adaptation results from improved growth in suspension rather than evasion of dilution [6]. This feature is critical for maintaining a well-mixed evolving population under defined selective conditions over extended periods. Importantly, active elimination of biofilm formation has previously been shown to be essential for long-term adaptive evolution experiments in GM3 systems, preventing cells from escaping selective pressure through wall growth [22].

**Fig. 1:**
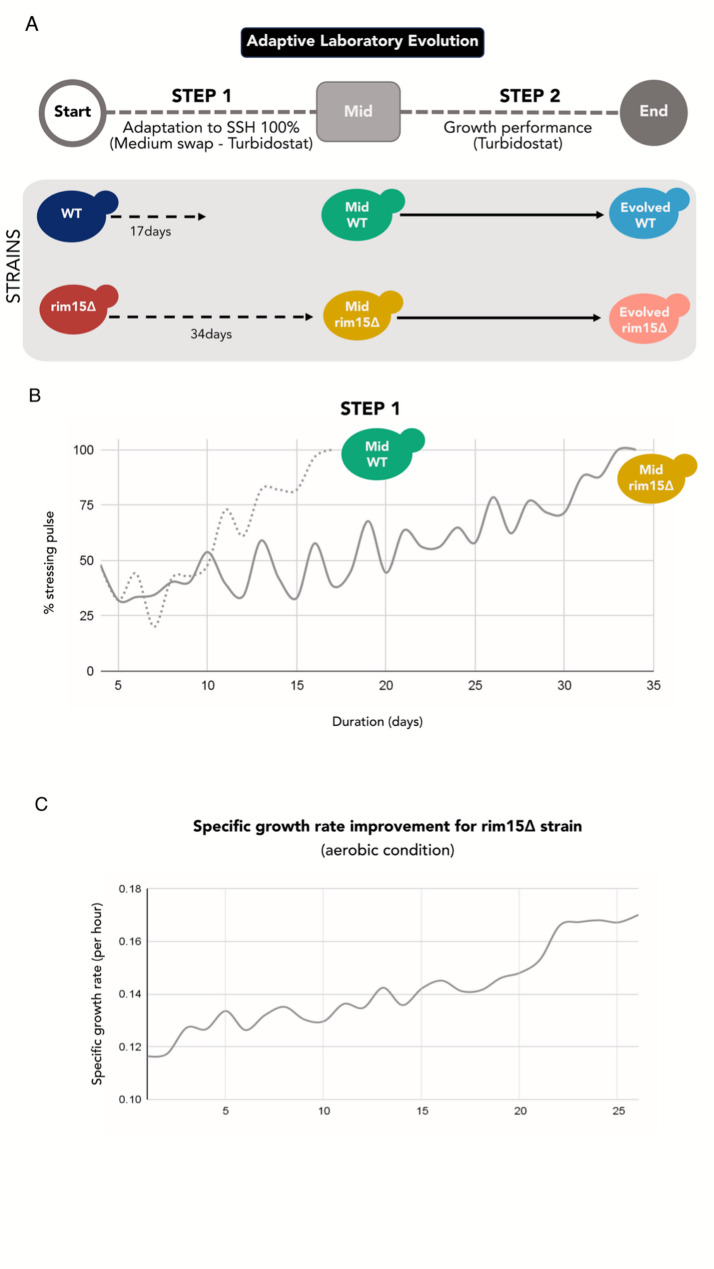
Adaptive laboratory evolution of *S. cerevisiae* strains in synthetic spruce hydrolysate (SSH). **A** Schematic representation of the adaptive laboratory evolution (ALE) setup used for the WT and rim15Δ strains. **B** Adaptation to 100% SSH under microaerobic conditions using a medium swap regime. The culture was periodically diluted either with relaxing medium (20% SSH) or stressing medium (100% SSH) as a function of transparency threshold (i.e., generation time). The y-axis (% stressing pulse) denotes the percentage of dilution pulses over a 24-h period, in which the stressing medium (100% SSH) was injected. **C** Growth rate improvement in 100% SSH under aerobic conditions for the rim15Δ strain cultivated in a turbidostat regime

The entire ALE experiment consisted of two sequential steps (Fig. 1A). In STEP1, cells were adapted to 100% SSH under microaerobic conditions using a medium-swap selective regime (Fig. 1B). In this regime, cultures were diluted every 10 min with either a relaxing medium (20% SSH) or a stressing medium (100% SSH), depending on a predefined transparency threshold linked to the target generation time. Because this threshold was kept constant, the system continuously maintained the population near its growth limit, thereby imposing a dynamic selective pressure that progressively increased the exposure to the stressing medium as adaptation proceeded. The observed oscillations (Fig. 1B) reflect the intrinsic feedback of the regime, in which population density determines the alternation between relaxing and stressing conditions.

The CEN.PK113-7D wild-type (WT) population reached full adaptation to 100% SSH within 17 days, whereas the rim15Δ strain required 34 days under identical conditions (Fig. 1B), indicating a reduced adaptive capacity in the absence of this central stress-response regulator. The adapted strains obtained at the end of STEP1 are referred to as midpoint clones: Mid WT and Mid rim15Δ.

In STEP2, growth performance was measured in 100% SSH under aerobic and microaerobic conditions. This step was essential to decouple transient physiological adaptation from stable genetic changes by evaluating growth performance under fixed conditions. The final strains isolated from this process are referred to as Evolved WT and Evolved rim15Δ. Considering that the specific growth rate of Mid rim15Δ was lower under aerobic than microaerobic conditions (Figure S1), an additional step was implemented in which the strain was cultivated aerobically in 100% SSH under a turbidostat regime for one month (Fig. 1C). This continuous selective pressure improved the specific growth rate by 1.34-fold, from 0.13/h to 0.17/h (Fig. 1C).

Adaptation of cells derived from the parental rim15Δ strain to 100% SSH suggests that canonical stress-response pathways are not strictly required for long-term adaptation to this lignocellulosic environment. Nevertheless, the fact that WT cells adapted twice as fast indicates that *RIM15* may facilitate a rapid and efficient stress response in this context. Rather than preventing adaptation entirely, deletion of *RIM15* appears to constrain the accessibility or efficiency of adaptive trajectories under lignocellulosic stress.

A second independent ALE trajectory was initiated for the rim15Δ strain using the same experimental setup and selection regime. Despite multiple reinoculation attempts and adjustments to cultivation parameters (including generation time and population size), this replicate population repeatedly failed to establish stable growth under 100% SSH and ultimately collapsed under the imposed selective pressure. These observations suggest that the accessible evolutionary trajectories in the rim15Δ background are more constrained, potentially requiring rare or specific mutational paths to achieve comparable fitness gains.

### Adaptive evolution increases yeast robustness across multiple perturbations

**Fig. 2:**
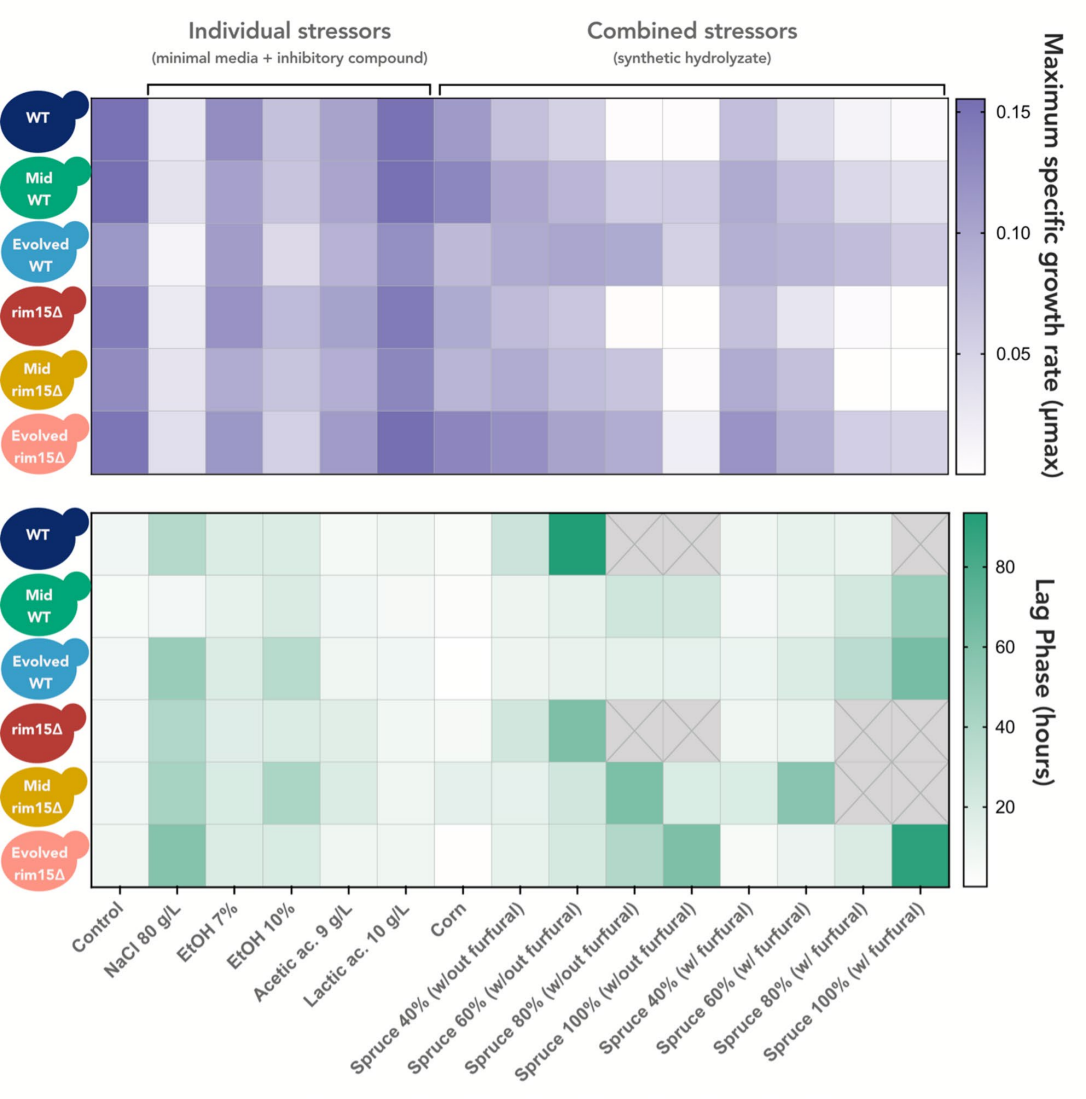
Performance of the parental and adapted strains in different perturbation conditions. The maximum specific growth rate (µmax; 1/h) and lag phase (h) of different strains (y-axis) are plotted against a variety of perturbation conditions commonly found in lignocellulosic hydrolysates (x-axis). Grey tiles with X: cultures did not grow

To evaluate the impact of ALE on strain performance, samples collected at different time points during evolution (Fig. 1A) were cultivated under 15 different perturbation conditions, including complex lignocellulosic hydrolysate and stressors commonly encountered in industrial fermentation processes. These perturbations represented different classes of industrial stresses, including osmotic stress (NaCl), organic acid stress (acetic and lactic acid), and ethanol stress. Maximum specific growth rate (µmax) and lag phase duration were measured and presented in Fig. 2; the parental strains were included as references (Fig. 2).

When cultivated in complex media containing synthetic lignocellulosic hydrolysates, evolved strains exhibited an increase in µmax and a reduction in lag phase relative to their corresponding parental strains. In contrast, no significant differences were observed when the strains were grown in minimal medium supplemented with a single inhibitory compound (Fig. 2). This suggests that the evolved phenotype is associated with the complex multifactorial stress environment imposed during ALE and is not fully reflected by the isolated inhibitory compounds tested herein.

**Fig. 3:**
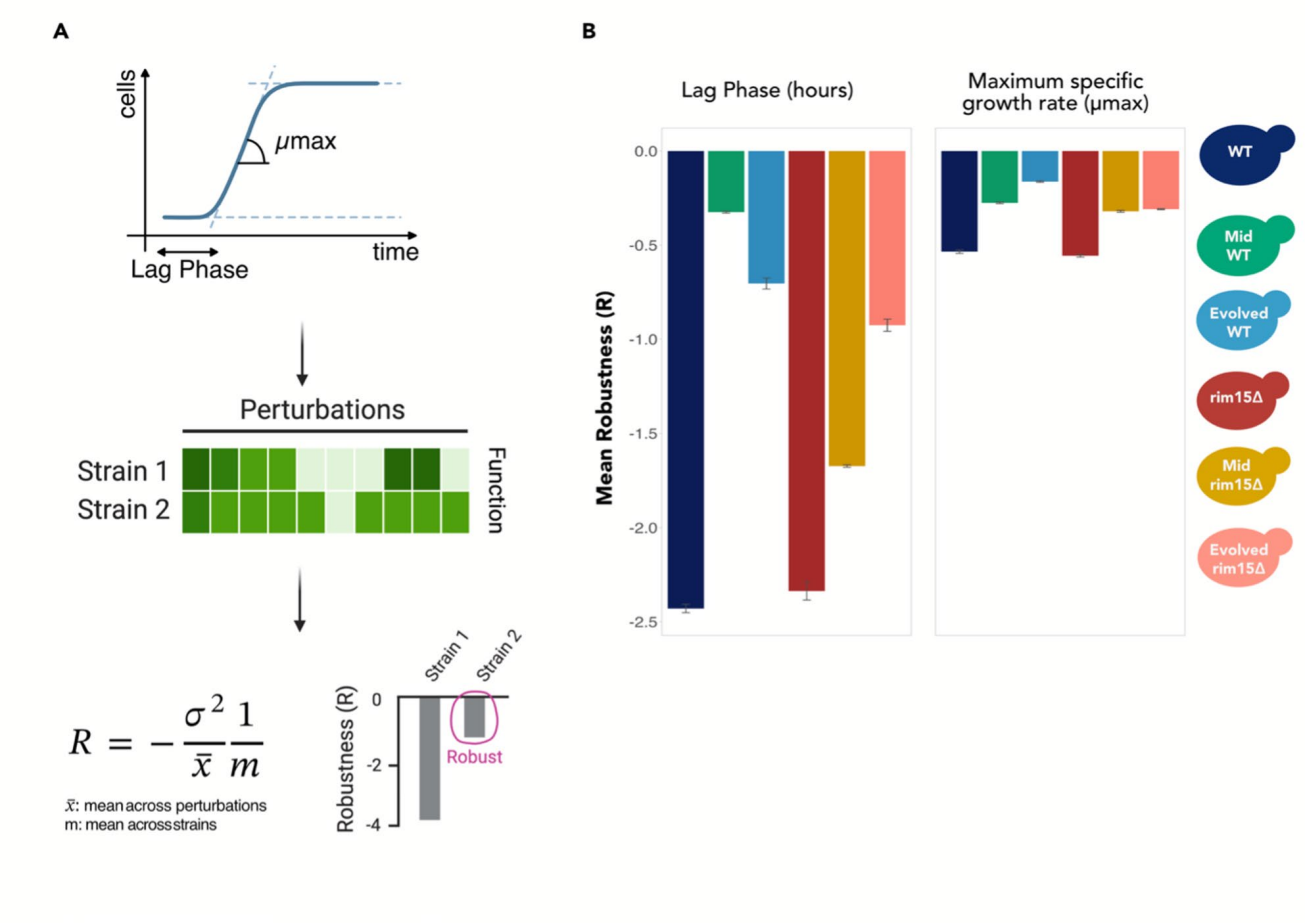
Robustness quantification of the parental and adapted strains in different perturbation spaces. **A** Schematic representation of the robustness calculation. Maximum specific growth rate (µmax) and lag phase were measured as functions for each strain across multiple perturbation conditions. Robustness was calculated as the normalized dispersion of each measurement across perturbations using Eq. 1 (described in Methods), where σ² represents the variance across perturbations, x̄ is the mean trait value across perturbations, and m is the mean across all strains. Values closer to zero indicate higher robustness across the tested perturbation space. **B** Robustness values calculated for µmax and lag phase for the parental, midpoint, and evolved strains. Error bars represent the standard deviation

To further investigate whether the improved phenotypes observed in Fig. 2 reflected increased microbial robustness, robustness was quantified for µmax and lag phase using Eq. 1, described in Methods and in Fig. 3A. As shown in Fig. 3B, both Evolved WT and Evolved rim15Δ displayed increased robustness for µmax and lag phase compared to their respective parental strains. These results indicate that adaptive evolution in SSH-generated strains results in more consistent phenotypic responses across the tested perturbation space, despite their distinct genetic backgrounds. Interestingly, robustness changes differed between the evaluated biological functions (µmax and lag phase), highlighting that robustness is function-dependent and may evolve differently depending on the physiological trait being analyzed.

Considering the higher robustness observed for the Evolved WT strain, its fermentation performance under microaerobic conditions was further evaluated in real lignocellulosic hydrolysates derived from different biomass sources. The parental WT and Evolved WT strains were cultivated in sugarcane, corn, and birch hydrolysates (composition described by Torello and collaborators [35], and metabolite concentrations were quantified after 48 h of fermentation (Figure S2). Under these conditions, the Evolved WT strain showed higher ethanol production compared to the parental WT strain across the tested hydrolysates. These results indicate that the Evolved WT strain displays improved fermentation performance under industrially relevant lignocellulosic conditions, highlighting the potential of robustness quantification as a metric for industrial strain selection.

### Divergent evolutionary strategies arise in WT vs. rim15Δ CEN.PK113-7D strains

To gain further insights into the molecular factors associated with adaptation of WT and rim15Δ strains during evolution, the DNA of different samples (parental, midpoint, and evolved) was extracted for whole-genome sequencing. To strengthen the genomic characterization, both bulk populations (overnight cultures representing the entire evolved population) and four isolated colonies per sample were sequenced, enabling assessment of population-level and clonal genetic variation arising during evolution.

**Fig. 4:**
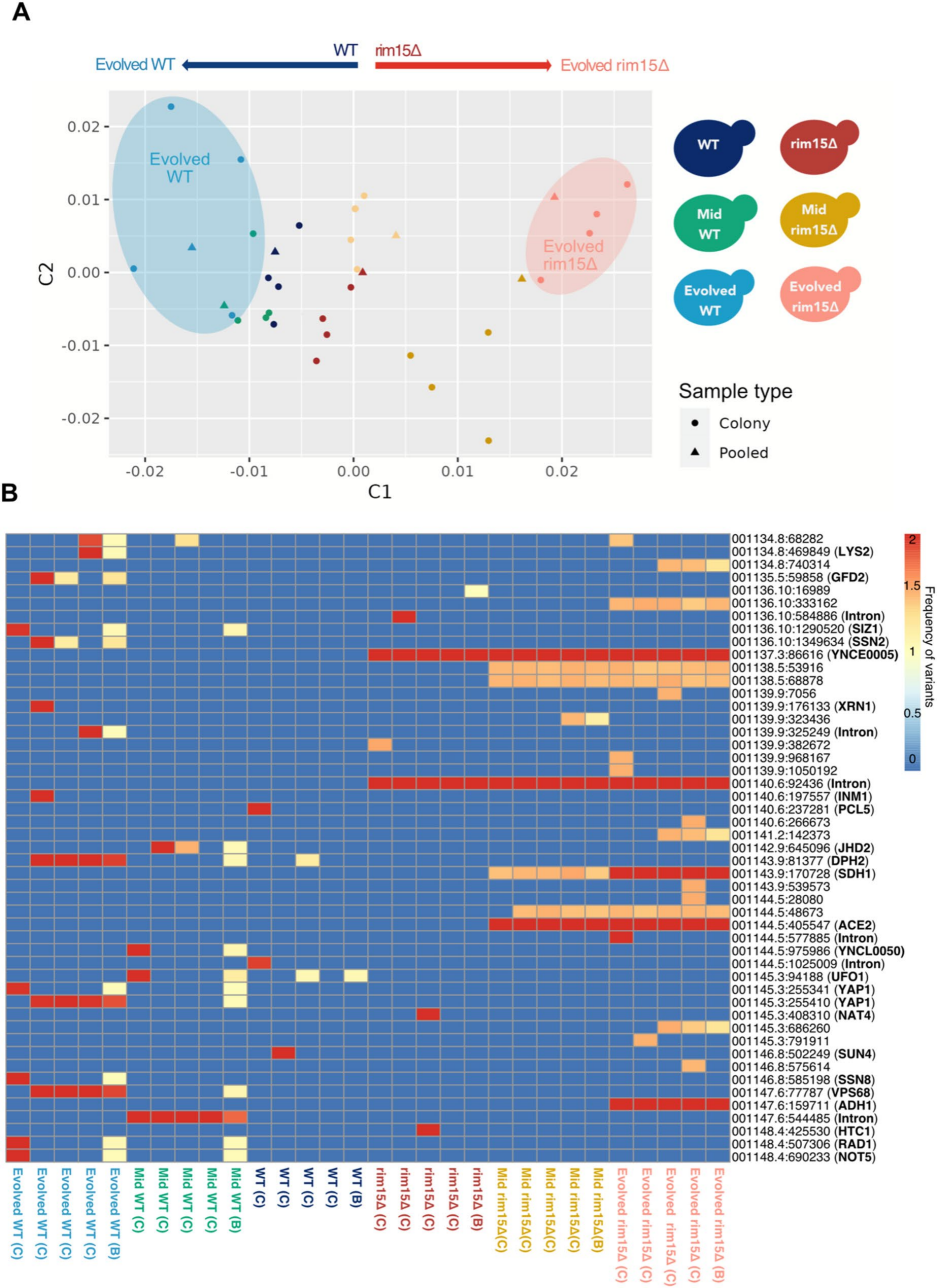
Genome-wide genetic variation across evolutionary stages of *S. cerevisiae* strains. **A** Multidimensional Scaling (MDS) analysis based on pairwise genetic distances among WT and rim15Δ strains at different evolutionary stages (parental, mid, and evolved). C1 and C2 represent the first and second MDS components. The plot highlights genotype-driven separation between WT and rim15Δ strains during evolution. Each point represents a sequenced sample from either an individual colony isolate or a bulk population. **B** Heatmap of variant distribution across parental, midpoint, and evolved strains. The x-axis denotes the strain name, bulk populations (**B**), and individual colony isolates (**C**). The y-axis denotes each detected variant with its corresponding chromosomal position and annotated gene for fixed mutations. Colors indicate the presence and frequency of variants: blue represents absence (reference allele), while yellow to red indicates increasing variant allele frequency, with red indicating fixation of the mutation

Figure 4A shows multidimensional scaling (MDS) analysis of the detected variants across all sequenced samples, including bulk populations and individual colonies. Evolved WT and Evolved rim15Δ strains separated into two distinct clusters, highlighting their different evolutionary trajectories despite being exposed to the same selective pressure, 100% SSH (Fig. 4A). This genotype-specific response indicates that the two strains accumulated different mutations, reflecting divergent adaptation strategies.

Evolved WT and Evolved rim15Δ strains diverged gradually from the parental strains, taking distinct and non-overlapping directions (Fig. 4A). While some intra-population variation existed between colonies and bulk from the same strain, these differences were less pronounced than those observed between evolved and parental strains, as revealed by MDS clustering.

The distribution and allele frequency of the detected variants across all sequenced samples are summarized in Fig. 4B. The heatmap reveals distinct mutation patterns between WT and rim15Δ strains across different evolutionary stages. Several variants increased in frequency or reached fixation within specific strains, with fixed mutations indicated in red in the heatmap. The combined analysis of bulk populations, isolated colonies, and allele frequency dynamics strengthened the identification of mutations associated with adaptation and enabled discrimination between fixed and colony-specific variants across the evolving populations. These allele frequency patterns provide the basis for calculating pairwise genetic distances between samples used in MDS analysis (Fig. 4A).

Fixed variants identified in bulk and single-colony samples are described in Table S1. The discussion below focuses on mutations in genes with previously described roles in stress adaptation or regulatory processes relevant to the phenotypes observed in this study. Variants detected in the Evolved WT strain appeared in genes associated with transcriptional regulation (*SSN2*, *SSN8*, and *NOT5*), stress response (*YAP1*), and mRNA turnover (*XRN1*).

**Fig. 5:**
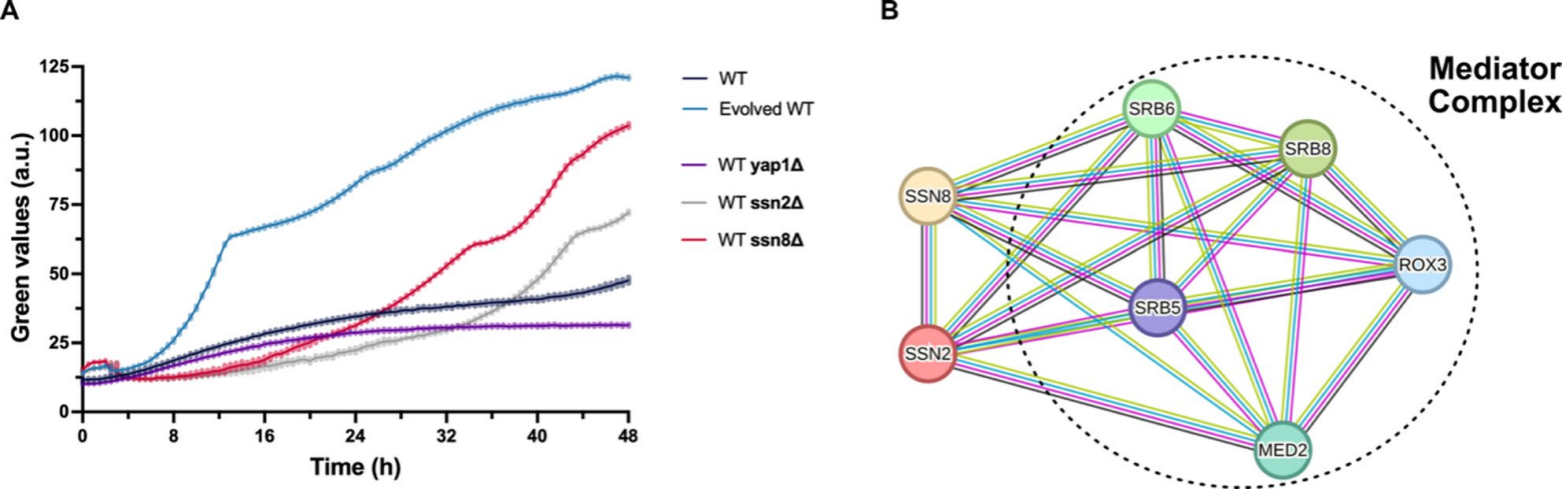
Growth phenotype of regulatory mutants and interaction network of mediator complex proteins. **A** Growth curves of the parental WT, Evolved WT strains and deletion mutants yap1Δ, ssn8Δ, and ssn2Δ cultivated under SSH 80%. Cells were grown in a Growth Profiler system (described in methods), and growth was monitored over time by measuring green value. The Evolved WT strain displayed the highest growth rate (µmax = 0.175 h⁻¹) and a short lag phase (~ 0.5 h), while the parental WT showed a lower growth rate (µmax = 0.028 h⁻¹). Deletion mutants ssn8Δ and ssn2Δ exhibited reduced growth rates (µmax = 0.058 h⁻¹ and 0.042 h⁻¹, respectively) and extended lag phases (~ 21.5 h and ~ 26 h, respectively). The yap1Δ mutant showed severely impaired growth (µmax = 0.009 h⁻¹). Error bars represent the standard deviation of three biological replicates. **B** Protein–protein interaction network generated using the STRING database showing interactions among mediator complex components (Srb5, Srb6, Srb8, Med2, and Rox3) associated with the regulatory pathway *SSN2* and *SSN8*. Nodes represent proteins and edges indicate predicted or experimentally supported functional associations derived from evidence sources. The network contains 7 nodes and 21 edges, the average node degree is 6, and the local clustering coefficient is 1, indicating a highly interconnected module. The protein–protein interaction enrichment p-value (9.87 × 10⁻⁸) indicates that the observed interactions are significantly higher than expected by chance

In *YAP1*, two frameshift mutations were identified: an insertion of T at nucleotide position 1501 resulting in a premature stop codon (detected in colony 3), and a deletion of A at nucleotide position 1569, also leading to a frameshift and premature stop codon (detected in the bulk population and colonies 1, 2, and 4). *YAP1* encodes a key transcription factor involved in oxidative stress resistance and has been widely reported to play an important role in cellular stress responses [23]. However, when the corresponding knockout mutant was grown in 80% of SSH, it exhibited reduced specific growth rate compared to the WT strain (Fig. 5A), suggesting that loss of *YAP1* function alone is insufficient to explain the evolved phenotype.

In contrast, deletion mutants of *SSN2* and *SSN8* showed improved growth rates in SSH compared to the WT strain, with higher growth rates (µmax = 0.058 h⁻¹ and 0.042 h⁻¹, respectively) than the parental WT (µmax = 0.028 h⁻¹; Fig. 5A). Both genes encode a subunit of the CDK8 kinase module (CKM) and are highly interconnected to a regulatory cluster of the Mediator complex (Fig. 5B), a regulatory subcomplex involved in transcriptional control and stress-response signaling [9]. Mechanistically, the CKM represses transcription of stress-responsive genes by preventing the interaction between the Mediator complex (Fig. 5B) and RNA polymerase II, whereas dissociation of this module relieves repression and allows transcriptional activation under stress conditions [3, 9]. Notably, these mutations emerged in the WT background, which retains a functional *RIM15* pathway previously linked to CKM regulation [42]. Therefore, alterations in the CKM-mediated transcriptional regulation may have contributed to adaptation in the WT lineage. Importantly, both variants were detected only in the Evolved WT population and were absent from the Mid WT population, indicating that they are unlikely to explain the initial increase in robustness observed during STEP1 and may instead have contributed to the later stages of adaptation during ALE.

The Evolved rim15Δ strain, instead, exhibited mutations in *SDH1* and *ACE2*, both of which were also present in the midpoint rim15Δ population and therefore likely fixed during evolution. *SDH1* encodes a subunit of succinate dehydrogenase, a component of the mitochondrial respiratory chain. Sequence analysis of the identified *SDH1* variant showed that this SNP was silent and therefore did not alter the protein sequence.

*ACE2* encodes a TF involved in cell cycle regulation and daughter cell separation [38]. Although *ACE2* has also been associated with biofilm formation [38], the GM3 system used in this study actively prevents wall growth and biofilm establishment. Therefore, unlike the Evolved WT strain, the adaptive trajectory of the rim15Δ strain appears to have involved changes primarily related to morphogenesis and cellular organization.

Previous studies have shown that *RIM15* deletion can increase fermentation rates in yeast [15]. In this context, the Evolved rim15Δ strain also accumulated a mutation in the alcohol dehydrogenase gene *ADH1*. The identified *ADH1* mutation resulted in an amino acid substitution from tyrosine to cysteine relatively close to the catalytic site (Figure S3A). Although the functional consequences of this mutation remain unclear without enzymatic assays, fermentation experiments in defined medium showed that the Evolved rim15Δ strain produced similar amounts of ethanol as the WT strain (Figure S3B).

A variant in the YNCE0005W tRNA gene was detected in all rim15Δ populations, indicating that this mutation may have arisen as a consequence of the *RIM15* deletion process rather than adaptive evolution itself.

Overall, the Evolved WT strain accumulated mutations primarily in genes related to stress response and transcriptional regulation; whereas the Evolved rim15Δ strain exhibited mutations associated mainly with morphogenetic processes. These results suggest that, even when subjected to the same selective pressure, different strains may adopt distinct adaptive strategies, potentially shaped by their parental genetic makeup. Extending this approach to other mutants could help identify recurrently targeted genes and pathways, offering further insight into general principles of adaptive evolution under stress.

### Multicellular aggregation in Evolved rim15Δ strains is driven by ACE2 mutation

**Fig. 6:**
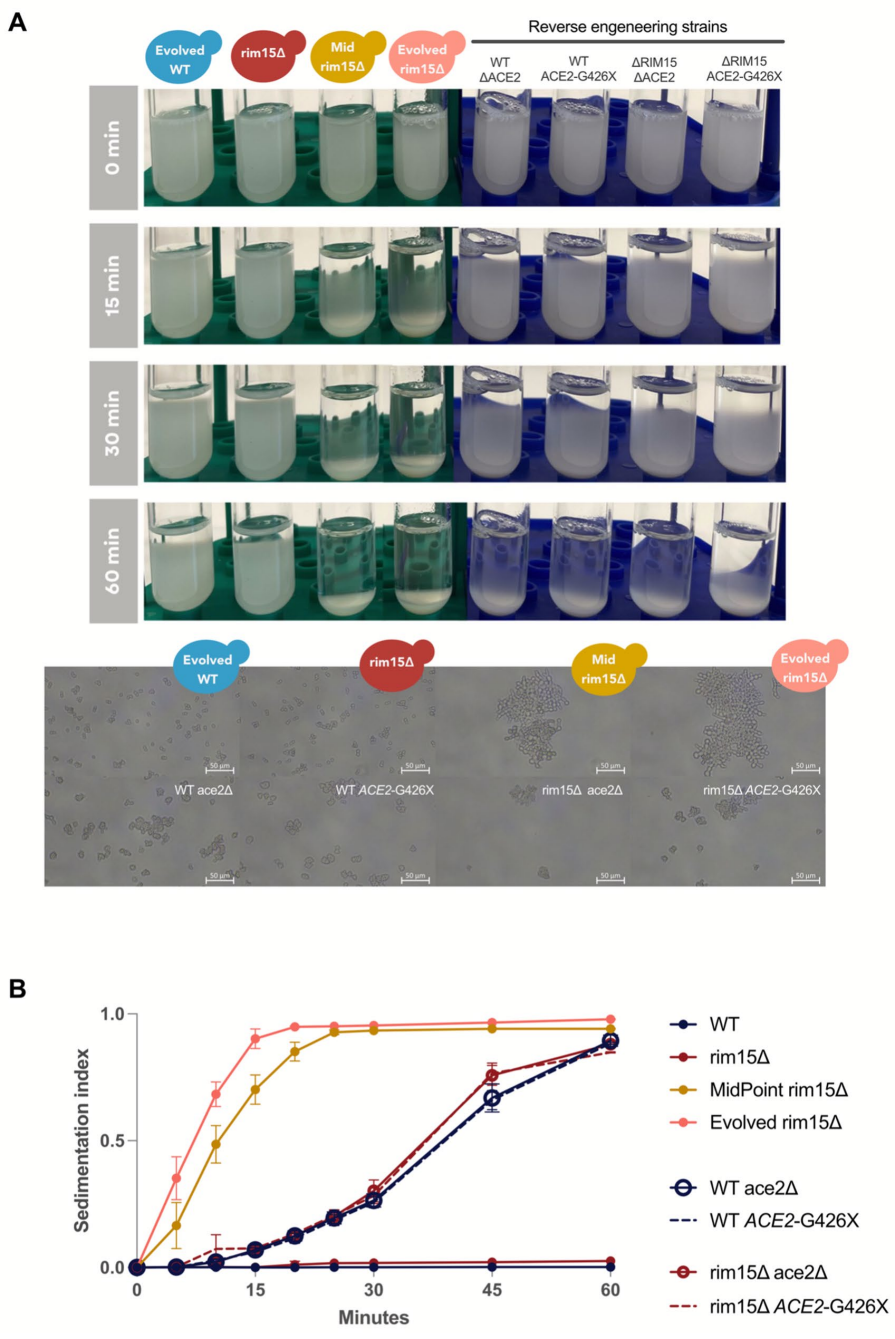
Sedimentation index and clump formation in parental, evolved, and engineered strains. **A** Visual assessment of cell sedimentation cultivated in defined minimal medium in test tubes at 0, 15, 30 and 60 min. Aggregation and cell settling are visible in Evolved rim15Δ and midpoint strains, with intermediate settling observed in reverse engineering strains (ace2Δ, *ACE2*-G426X, rim15Δ-ace2Δ, and rim15Δ-*ACE2*_G426X). Brightfield microscopy (40×) images (scale bar: 50 μm) of the same strains showing cell morphology and clump formation. **B** Sedimentation index (OD_600initial_-OD_600timepoint_ /OD_600initial_) measured over 60 min for rim15Δ parental, midpoint, and evolved strains, as well as reverse-engineered strains (ace2Δ, *ACE2*-G426X, rim15Δ-ace2Δ, and rim15Δ-*ACE2*_G426X). Error bars represent the standard deviation of three biological replicates

During ALE, the rim15Δ strain developed a sedimentation phenotype, with visible cell aggregation detectable already in the midpoint population (Fig. 6A). Microscopy and visual analysis revealed impaired cell separation and the formation of dense multicellular clumps. Attempts to disperse the clumps using EDTA and mannose cultivation were unsuccessful, suggesting that the aggregation was not solely due to calcium-dependent flocculation and might involve additional mechanisms beyond the canonical flocculation pathway [33].

Whole-genome sequencing of the Evolved rim15Δ strain identified a nonsense mutation in *ACE2* (G426X), a TF regulating daughter-specific genes involved in septum degradation during mitotic exit (Saccharomyces Genome Database ID: S000004121; YLR131C). A single point mutation in this gene has been observed in previous ALE experiments with sequential batch reactors [13, 26], suggesting that *ACE2* is a likely target under certain evolutionary selective pressures. In the context of this study, the fact that only the rim15Δ lineage, and not the WT, acquired the *ACE2* mutation under identical selective conditions suggests a possible link between *RIM15* disruption and the advantage offered by a clump-forming phenotype. Previous studies have proposed that multicellular aggregation can enhance survival under environmental stress by creating protective microenvironments that reduce individual cell exposure to toxic compounds and facilitate cooperative stress responses [4]. Therefore, clump formation may represent an alternative adaptive strategy that contributes to persistence under lignocellulosic stress in the absence of a functional *RIM15* pathway.

*ACE2* inactivation in the rim15Δ strain may reflect a compensatory adaptation strategy (Figure S4). *RIM15* functions as a central integrator of nutrient and stress signals via the protein kinase A and target of rapamycin pathways, coordinating entry into quiescence and sporulation through downstream effectors, such as *IME1* and *MSN2/4* [34]. As shown in prior studies with *TAO3*, involved in regulation of Ace2 activity and cellular morphogenesis [12], disruption of developmental regulators can shift evolutionary trajectories toward altered morphogenetic states. In the absence of a functional *RIM15*, clump formation may offer a favorable alternative to cope with sustained lignocellulosic stress, enhancing tolerance through microenvironmental buffering, resource sharing, and protection from inhibitors [13, 28].

To assess the functional relevance of the *ACE2* mutation, parental WT and rim15Δ were reverse-engineered to yield two genetically modified strains: one harboring a complete deletion of *ACE2* (ace2Δ and rim15Δ-ace2Δ), and another carrying the specific G426X mutation identified in the Evolved rim15Δ genome (*ACE2*_G426X and rim15Δ-*ACE2*_G426X). All engineered strains exhibited increased sedimentation and cell aggregation compared to WT and rim15Δ, confirming that *ACE2* loss-of-function contributed significantly to the phenotype (Fig. 6B). However, neither engineered strain fully reproduced the sedimentation kinetics observed in the evolved lineage, indicating that the *ACE2* mutation alone does not fully explain the phenotype. Similar observations have been reported in a previous study [26], whereby *ACE2* mutants formed multicellular aggregates but did not necessarily exhibit rapid settling behavior, explained by differences in ploidy that affected cell size and sedimentation dynamics. In this work, both in silico analyses and flow cytometry were inconclusive regarding the ploidy status of the evolved strains. For flow cytometry, it was difficult to dissociate cell clumps and obtain reliable single-cell measurements. Additionally, microscopic observations revealed morphological heterogeneity within the Mid rim15Δ and Evolved rim15Δ populations, including the presence of both larger and smaller cells, which may suggest alterations in ploidy. However, confirmation of this hypothesis would require single-cell analyses, which are beyond the scope of the present study.

## Conclusion

Our study demonstrates that deletion of the central stress integrator *RIM15* does not prevent adaptive evolution in lignocellulosic hydrolysates. Using two-step ALE in SSH, we show that both WT and rim15Δ strains acquired increased robustness, although over different physiological and genetic strategies. Notably, the Evolved rim15Δ strain adopted a morphogenetic adaptive strategy characterized by multicellular aggregation. Although ACE2 inactivation contributed substantially to this phenotype, additional evolutionary events likely shaped the final sedimentation behavior, potentially by promoting larger aggregates or changes in cellular morphology and ploidy. In contrast, the WT strain accumulated mutations mainly in genes involved in transcriptional regulation and stress-response pathways, including components of the Mediator complex. Altogether, our work provides new insights into regulatory and morphogenetic strategies employed by yeast to cope with hydrolysate stress and increase robustness and offers new avenues for engineering strains tailored for harsh bioprocess conditions. Moreover, our findings highlight the value of leveraging evolved strains from industrial contexts to uncover novel adaptive mechanisms and guide future engineering efforts.

## Materials and methods

### Strain maintenance

The strains used in this work are described in Table 1. All strains were derived from *S. cerevisiae* CEN.PK113-7D. Stocks were prepared from shake flask cultures grown in YPD medium (10 g/L yeast extract, 20 g/L peptone, and 20 g/L glucose) at 30 °C and shaken at 200 rpm. Glycerol was added (16% v/v final concentration), and the stocks were stored at -80 °C.

**Table 1:** Strains used in this work

Name in this study	Genotype	Source
WT	*S. cerevisiae* CEN.PK113-7D (MATa, URA 3, HIS3, LEU2, TRP1, MAL2-8c and SUC2)	Scientific Research and Development GmbH (Oberursel, Germany)
Mid WT	*S. cerevisiae* CEN.PK113-7D after STEP 1 of ALE	This study
Evolved WT	*S. cerevisiae* CEN.PK113-7D after STEP 2 of ALE	This study
rim15△	*S. cerevisiae* CEN.PK113-7D rim15△	This study
Mid rim15△	*S. cerevisiae* CEN.PK113-7D rim15△ after STEP 1 of ALE	This study
Evolved rim15△	*S. cerevisiae* CEN.PK113-7D rim15△ after STEP 2 of ALE	This study
yap1△	*S. cerevisiae* CEN.PK113-7D yap1△	This study
ssn2△	*S. cerevisiae* CEN.PK113-7D ssn2△	This study
ssn8△	*S. cerevisiae* CEN.PK113-7D ssn8△	This study
ace2△	*S. cerevisiae* CEN.PK113-7D ace2△	This study
*ACE2*_G246X	*S. cerevisiae* CEN.PK113-7D *ace2_G246X*	This study
rim15△-ace2△	*S. cerevisiae* CEN.PK113-7D rim15△, ace2△	This study
rim15△-*ACE2*_G246X	*S. cerevisiae* CEN.PK113-7D rim15△, *ace2_G246X*	This study

### Culture conditions

ALE experiments were performed in SSH, which was designed to mimic the major inhibitory characteristics of spruce hydrolysate while remaining compatible with turbidity-based growth measurements. The composition of SSH was based on the literature and is listed in Table 2. For experiments using 20% SSH, the hydrolysate was diluted in defined minimal medium containing 5 g/L (NH₄)₂SO₄, 3 g/L KH₂PO₄, 1 g/L MgSO₄·7 H₂O, 20.4 g/L potassium phthalate buffer, 0.5 mL/L trace mineral solution (Table S2), and 0.5 mL/L vitamin solution (Table S2). The pH of the medium was adjusted to 5.0 and the complete medium was sterilized by filtration.2

To assess µmax and lag phase under various stress conditions, the same minimal medium was used to dilute SSH to a final concentration of 40%, 60%, and 80% (v/v). Minimal medium supplemented with 20 g/L glucose was used for cultivation in the presence of the following stressors commonly found in industrial processes: 80 g/L NaCl, 7% (v/v) ethanol, 10% (v/v) ethanol, 9 g/L acetic acid, or 10 g/L lactic acid. The detailed composition of synthetic spruce hydrolysates used in the assay is listed in Table 2.

**Table 2:** The composition of synthetic spruce hydrolysates (SSH) used in this study

Chemical (CAS number)	(g/L)
D-(+)-Glucose (14431-43-7)	34
D-(+)-Xylose (58-86-6)	9
L-(+)-Arabinose (5328-37-0)	4
D-(+)-Galactose (59-23-4)	4.50
Acetic acid (64-19-7)	7.50
Levulinic acid (123-76-2)	2.40
Furfuryl alcohol (98-00-0)	1.10
5-(Hydroxymethyl)furfural (67-47-0)	3.40
Vanillin (121-33-5)	0.11

Forthe ALE experiments, furfuryl alcohol was used instead of furfural due tooperational restrictions associated with the evolution platform and was addedat the same concentration as for furfural. Compound compositions arerepresented in g/L

### Adaptative laboratory evolution

The ALE experiment was carried out in a GM3 automat, a dual-chamber continuous culture device that performs a cleansing cycle with 5 M NaOH every 12 h to prevent biofilm formation [22]. Microaerobic conditions were achieved by flushing the chambers with 100% N_2_ gas. Because the cultures alternated between chambers, some residual oxygen remained in the environment, making it impossible to ensure strictly anaerobic conditions.

In STEP1, a medium swap selective regime was used under microaerobic conditions, with 100% SSH as the stressing medium, 20% SSH as the relaxing medium, and 4.5 h as the minimum imposed generation time. Every 10 min, a pulse of medium was injected into the culture chamber based on the transparency threshold. If the transparency was above the threshold (indicating lower cell density), a dilution pulse of relaxing medium (20% SSH) was applied; if the transparency was below the threshold (indicating higher cell density), a dilution pulse of stressing medium (100% SSH) was applied. The percentage of stressing pulses (% stressing pulse) was calculated as the fraction of dilution events corresponding to injections of stressing medium relative to the total number of dilution pulses over a 24-h period.

Once cell densities in the stressing medium stabilized below the transparency threshold for multiple days, the cells were considered adapted to 100% SSH. In STEP2, the maximum specific growth rate of both strains was increased in a regular turbidostat regime using 100% SSH for cultivation. Evolution continued until the cultures achieved the same generation time observed during cultivation with 20% SSH.

### High-throughput growth profiling

Growth performance under different perturbation conditions was evaluated using a high-throughput cultivation platform adapted from Trivellin et al. [36].

Overnight cultures were prepared in defined minimal medium containing 20 g L⁻¹ glucose and incubated at 30 °C with shaking at 200 rpm. Exponentially growing cultures were used to inoculate square 96-half-deepwell microtiter plates (Enzyscreen) at an initial OD_600_ of 0.02. Each well contained one perturbation condition, and all conditions were evaluated in biological triplicates. The tested perturbation space included different concentrations of synthetic spruce hydrolysate (SSH; 40%, 60%, 80%, and 100%) as well as defined minimal medium supplemented with individual industrial stressors (NaCl, ethanol, acetic acid, or lactic acid), as described in Sect. “Culture conditions”.

Microplates were sealed with CO₂-release covers (Enzyscreen) to minimize oxygen transfer and cultivated in a Growth Profiler 960 (Enzyscreen) at 30 °C with orbital shaking at 250 rpm for 48 h. Cell growth was monitored automatically throughout cultivation by measuring the green value (GV), a digital image-derived parameter proportional to biomass accumulation. Growth curves were exported and analyzed in R. Maximum specific growth rate (µmax) was estimated from spline-fitted growth curves using the all_splines function. At the same time, lag phase duration was calculated as the intersection between the tangent at the inflection point and the horizontal line corresponding to the initial biomass, as previously described by Trivellin et al. [36].

For wells in which no detectable growth occurred during the cultivation period, µmax was assigned a value of zero and lag phase was recorded as not available (NA). The resulting µmax and lag phase values were subsequently used to quantify robustness, as described in Sect. “Robustness quantification”.

### Robustness quantification

Robustness was quantified using Eq. 1 as described previously [36]. This equation was selected over other dispersion measures, such as 1-CV [43] and Kitano’s method [16], because it is dimensionless, control-free, and accurately represents the robustness of biological traits across different orders of magnitude. The equation quantifies the index of dispersion $\left(\frac{{\sigma\:}^{2}}{\stackrel{-}{x}}\right)$ across a dataset and normalizes the value with respect to the mean (m) across all perturbations and strains. Robustness is a negative value, where 0 is the maximum achievable. The robustness of each strain for a specific function (µmax or lag phase) was computed across multiple perturbations.

1 $$ R = - \frac{{\sigma ^{2} }}{{\bar{x}}}*\frac{1}{m} $$

### Whole-genome sequencing

Both bulk and isolated colonies were subjected to whole-genome sequencing to assess the genomic alterations arising during ALE. For that, samples collected during ALE were first cultivated in 10 mL YPD medium (Fig. 1A) at 30 °C overnight; this culture constituted the bulk sample. From these overnight cultures, aliquots were plated onto YPD agar plates and incubated at 30 °C for 24 h to isolate individual colonies. Four single colonies per sample were selected and grown in 10 mL YPD medium at 30 °C overnight to obtain sufficient biomass for DNA extraction.

Genomic DNA was extracted using the Quick-DNA™ Fungal/Bacterial Miniprep Kit (Zymo Research), following the manufacturer’s protocol. Cell lysis was performed using a bead-beating procedure with the following parameters: 4 cycles at 4500 rpm for 1 min each and 2 min rest intervals between cycles. DNA concentration and quality were assessed using 1% (w/v) agarose gel electrophoresis, Nanodrop spectrophotometry, and Qubit fluorometric quantification.

Library preparation was performed using the Illumina DNA PCR-Free protocol, and paired-end sequencing (2 × 150 bp) was carried out by the National Genomics Infrastructure in Stockholm, part of the Science for Life Laboratory. Quality control of raw sequencing reads was performed using FastQC (v0.11.9) and FastQ Screen (v0.14.1), with summary visualization through MultiQC (v1.13).

### Mapping/variant calling

All sequencing reads were trimmed using Cutadapt (v. 4.0) to remove adapter sequences. Reads from two lanes were mapped separately to the *S. cerevisiae* reference sequence [GCF_000146045.2_R64] using BWA-MEM (v. 0.7.17) with default settings. The resulting BAM files were sorted using SAMtools (v. 1.9). BAM files corresponding to each sample were then merged, and duplicate reads were marked using MarkDuplicates (GATK v4.1.4.1). Variant calling was performed with FreeBayes (v. 1.3.2) using the following parameters: flags: -0 -C 4 -f 0.2 --use-best-n-alleles 4.

### Variant filters and annotation

For variant filters, VCF files generated by FreeBayes were processed using vcftools (v. 0.1.14). Variants with low quality (QUAL < 20), high missingness (> 20%), low (< 100) or high (> 400) mean depth, genotypes of low depth (< 30), and multi-allelic sites were removed using the parameters: vcftools --minQ 20 --max-missing 0.8 --min-meanDP 100 --max-meanDP 400 -minDP 30 --min-alleles 2 --max-alleles 2. Additionally, variants fixed in all samples were excluded.

Variants were annotated using Variant Effect Predictor (110.1), with default settings, except for the use of “--distance 100” and the addition of the “--sift b” parameters.

### Multidimensional scaling

PLINK2 (v. 2.00-alpha-2.3-20200124) was used to convert the VCF file to PLINK format using the following parameters: --vcf < vcf> --allow-extra-chr --set-all-var-ids @:# --vcf-half-call m --make-bed. MDS analysis was performed with PLINK (v. 1.90 b4.9) following LD-pruning of the variants using --indep 50 5 2. For this calculation, the *RIM15* deletion (NC_001138.5) was excluded.

### Sedimentation assay

 Cells were cultivated for 36 h at 30 °C in baffled flasks containing 7 mL of defined minimal medium supplemented with 20 g/L glucose. After cultivation, the cells were harvested (4000 rpm, 10 min), and 150 mg cell pellet/strain was resuspended in 5 mL phosphate-buffered saline (137 mM NaCl, 2.7 mM KCl, 10 mM Na₂HPO₄, 1.8 mM KH₂PO₄, pH 7.4). Of these 5 mL, 1 mL was used for OD_600_ measurement with a PV4 spectrophotometer (VWR^®^), and 4 mL was used to visualize cell sedimentation in a test tube. OD_600_ was recorded continuously for 30 min, and pictures were taken at time zero and after 30 min. The sedimentation index was defined as the ratio of the decrease in OD_600_ between each time point and the initial OD_600_ value: OD_600initial_ - OD_600timepoint_ / OD_600initial_ [26]. Results are presented as biological replicates.

### Construction of knockout strains

All strains constructed in this study derive from the laboratory CEN.PK113-7D strain (Table 1). Modifications to the genome (deletion or codon replacement) were carried out combining the LiAc/salmon sperm carrier DNA/polyethylene glycol method [10, 11] and CRISPR/Cas9 for improved integration efficiency [1]. The CRISPR-Cas9 expression system and design used in this study were developed in our laboratory [5]. Briefly, the YN2_1_Cas9 plasmid bore both the Cas9 and single guide RNA (sgRNA) expression cassettes. The sgRNA expression cassette was designed with a GFP-dropout insert, which was replaced by the gene-specific 20-bp sgRNA. The sgRNA sequences required for genome modifications and the donor DNAs needed for transformations were designed using an automated tool available from GitHub (https://github.com/lucatorep/sgRNA_design_scripts). Briefly, the script compares sgRNA candidates across three different websites: CRISPR-ERA [21], Yeast CRISPRi [32], and CHOPCHOP [18]. The most suitable sgRNAs are selected based on the following desired parameters: (I) ATAC-seq > 0.7; (II) nucleosome presence < 0.2; (III) absence of poly-nucleotide sequences (more than 4 identical nucleotides in a row) and off-targets; (IV) CG content > 25%; (V) sgRNA sequence present in multiple databases, and (VI) prediction of sgRNA efficiency > 50%.

To completely remove the coding sequence of the gene selected for deletion, an 11-bp sequence comprising three stop codons in different frames (TAACTAGCTGA) was flanked by 50-bp homology arms in the promoter and terminator regions of the selected gene. Homology arms were automatically selected from the R script mentioned above. Therefore, the final donor DNA was 111 bp long (Table S3).

All oligos for each gene (sgRNAs and donor DNAs) were ordered as single-stranded (forward and reverse) oligonucleotides from Eurofins. The sgRNAs already contained sticky ends suitable for assembly in the YN2_1_Cas9 vector (Table S3). The generation of double-stranded oligonucleotides (both sgRNAs and donor DNAs), along with insertion of sgRNA into YN2_1_Cas9 were carried out as described previously [5]. Yeast transformation [10, 11] required an 18-min heat shock, 1 µg double-stranded donor DNA, and 300 ng Cas9-sgRNA plasmid for each gene. Transformants were then plated on YPD + G418 (geneticin selection marker) plates and incubated for 3 days at 30 °C. Colonies were verified by colony PCR using gene-specific oligos. The Cas9 plasmid was cured by re-streaking positive clones twice on antibiotic-free YPD plates.

### Data analysis

Data analysis was carried out in RStudio (Version 2026.05.0 + 218) or GraphPad Prism (Version 10.1.1).

## Supplementary Information

Below is the link to the electronic supplementary material.

Supplementary Material 1.

## Data Availability

No datasets were generated or analysed during the current study.

## Abbreviations

ALE: Adaptive laboratory evolution
CKM: CDK8 kinase module
GM3: Continuous–culture device
MDS: Multidimensional scaling
SSH: Synthetic spruce hydrolysate
STRING: Functional protein association networks
TF: Transcription factor
WT: Wild–type
µmax: Maximum specific growth rate
