# Supplementary material for: Effects of RIM15 deletion on yeast adaptation to synthetic spruce hydrolysate
Source: Microb Cell Fact. 2026 Aug 15;25:169. doi: 10.1186/s12934-026-03096-6 (PMC13477587; doi:10.1186/s12934-026-03096-6)
Supplement: Supplementary file 1 [file 12934_2026_3096_MOESM1_ESM.docx]

# **Supplementary material**

**Figure S1. Specific growth rate per hour during adaptation.** WT and rim15Δ cells were grown in a turbidostat, and measurements of the specific growth rate per hour were taken under microaerobic and aerobic conditions.

**Figure S2. Metabolite concentrations after 48 h of fermentation by parental WT and evolved WT strains in different lignocellulosic hydrolysates.** The concentrations of consumed glucose (blue), glycerol (light purple), and ethanol (green) were measured by HPLC after fermentation of 60% hydrolysates of sugarcane, corn, and birch by the two WT strains. Fermentations were performed in shake flasks under microanaerobic conditions using water locks. The 48-h time point was selected for comparison because, at this time, both strains had fully consumed the glucose in the sugarcane hydrolysate. Ethanol yield $\left( Y_{EtOH/S} \right)$, calculated as grams of ethanol produced per gram of glucose consumed (g g⁻¹), is indicated next to each condition. The composition of the hydrolysates has been previously described (Torello Pianale *et al.*, 2023).

**Figure S3: Structural analysis of the Adh1 protein of parental rim15Δ and evolved rim15Δ strains. A)** The three-dimensional structures of the parental (rim15Δ) and evolved rim15Δ Adh1 proteins were predicted using AlphaFold2 (AlphaFold Protein Structure Database). The model with the highest pLDDT confidence score (ranked model 1) was selected for structural analysis. Whole-genome sequencing of the evolved strain identified a single nucleotide substitution at position 884 (A→C), resulting in a Tyr-to-Cys amino acid substitution at position 295 (Y295C) (indicated by the dashed black box in the figure). Catalytic residues (Cys43, His66, Glu67, and Cys153) are highlighted in orange, while residue 295 is shown in red. Structural visualization and distance measurements were performed using PyMOL (version 3.0.3). The Cα–Cα distances between residue 295 (mutation site) and catalytic residues were calculated to assess potential structural proximity to the active site. The measured distances were: 295–Cys43, 17.95 Å; 295–His66, 17.17 Å; 295–Glu67, 14.50 Å; and 295–Cys153, 13.56 Å. **B)** Growth and ethanol production of parental rim15Δ and evolved rim15Δ strains cultivated in defined medium containing 2% glucose. This medium was selected to allow the parental rim15Δ strain to grow at a similar specific growth rate to the evolved rim15Δ strain. Cells were cultivated in the Growth Profiler system and growth was monitored through green values. Ethanol concentrations in the culture supernatant were quantified by HPLC after 6 and 12 h of cultivation. Error bars represent the standard deviation of two biological replicates.

**Figure S4. Protein–protein interaction network and Gene Ontology (GO) enrichment analysis of Rim15 and RAM pathway components. A)** The interaction network was constructed using the STRING database (version 12.0; https://string-db.org) for *S. cerevisiae*. The query included Rim15 and core components of the Regulation of Ace2 and Morphogenesis (RAM) pathway, including Cbk1, Mob2, Kic1, Hym1, Tao3, and Ace2. The interaction sources considered were experimentally validated interactions, curated databases, co-expression, gene neighborhood, gene fusion, and text mining, using a minimum required interaction score of 0.4 (medium confidence). Disconnected nodes were hidden for clarity. The resulting network contained 9 nodes and 27 edges, with an average node degree of 6 and an average local clustering coefficient of 0.886, indicating a highly interconnected module. The expected number of edges was 0, and the PPI enrichment p-value was < 1.0 × 10⁻¹⁶, demonstrating that the observed interactions occurred significantly more frequently than expected for a random set of proteins of comparable size. This strongly supports functional connectivity within the network rather than random association. **B)** Gene Ontology (GO) enrichment analysis of the proteins included in the network. Functional enrichment was calculated using STRING’s built-in overrepresentation test against the whole *S. cerevisiae* genome background, applying false discovery rate (FDR) correction for multiple testing. The most significantly enriched biological process terms included cell separation, regulation of cell morphogenesis, protein phosphorylation, and stress response (FDR < 0.05). Bubble size represents gene count within each GO term, while color intensity reflects statistical significance (−log10 FDR).

**Table S1. Gene annotations of fixed variants detected by whole-genome sequencing.** Variants are classified as SNP (single nucleotide polymorphism), del (deletion), or ins (insertion). Mutations were identified either during sequencing of the evolving population (Bulk) or in individual isolates (Colonies) obtained from the populations. Numbers in the Colonies column indicate the specific isolates in which the mutation was detected.

| **Strain** | **Gene** | **Type** | **Sequencing analysis** | |
| --- | --- | --- | --- | --- |
|  |  |  | **Bulk** | **Colonies** |
| WT | PCL5 | SNP | - | 3 |
|  | SUN4 | SNP | - | 2 |
| Mid WT | JHD2 | SNP | - | 2 and 4 |
|  | YNCL0050C | SNP | yes | 3 |
|  | UFO1 | SNP | yes | 3 |
| Evolved  WT | LYS2 | SNP | - | 4 |
|  | GFD2 | SNP | - | 2 |
|  | SIZ1 | SNP | - | 3 |
|  | SSN2 | SNP | - | 2 |
|  | XRN1 | SNP | - | 2 |
|  | INM1 | SNP | - | 2 |
|  | DPH2 | SNP | yes | 1, 2 and 4 |
|  | YAP1 | del and ins | yes | 1, 2, 3 and 4 |
|  | SSN8 | SNP | - | 3 |
|  | VPS68 | SNP | yes | 1, 2 and 4 |
|  | RAD1 | SNP | - | 3 |
|  | NOT5 | SNP | - | 3 |
| rim15Δ | YNCE0005W | SNP | yes | 1, 2, 3, and 4 |
|  | NAT4 | SNP | - | 2 |
|  | HTC1 | SNP | - | 2 |
| Mid  rim15Δ | YNCE0005W | SNP | yes | 1, 2, 3, and 4 |
|  | SDH1 | SNP | yes | 1, 2, 3, and 4 |
|  | ACE2 | SNP | yes | 1, 2, 3, and 4 |
| Evolved  rim15Δ | YNCE0005W | SNP | yes | 1, 2, 3, and 4 |
|  | SDH1 | SNP | yes | 1, 2, 3, and 4 |
|  | ACE2 | SNP | yes | 1, 2, 3, and 4 |
|  | ADH1 | SNP | yes | 1, 2, 3, and 4 |

**Table S2: Composition of trace metals and vitamin solutions used in the defined minimal medium.**

| **Solution** | **Chemical** | **Amount (g/L)** |
| --- | --- | --- |
| **Trace metals solution** | EDTA | 0.015 |
|  | ZnSO_4_∙7H_2_O | 0.0045 |
|  | MnCl_2_·4H_2_O | 0.0008 |
|  | CoCl_2_·6H_2_O | 0.0003 |
|  | CuSO_4_·5H_2_O | 0.0003 |
|  | Na_2_MoO_4_·2H_2_O | 0.0004 |
|  | CaCl_2_·2H_2_O | 0.0045 |
|  | FeSO_4_·7H_2_O | 0.003 |
|  | H_3_BO_3_ | 0.001 |
|  | KI | 0.0001 |
|  | **Vitamin** | **Amount (g/L)** |
| **Vitamin solution** | d-Biotin | 0.00005 |
|  | Calcium D(+) pantothenate | 0.001 |
|  | Nicotinic acid | 0.001 |
|  | Myo-inositol | 0.025 |
|  | Thiamine HCl | 0.001 |
|  | Pyridoxine HCl | 0.001 |
|  | Para-aminobenzoic acid | 0.0002 |

**Table S3:** **Oligonucleotide sequences used in this study.** The listed oligonucleotides were annealed and used either as donor DNA or as sgRNA templates for gene deletion or truncation (as indicated in the oligo code). Lowercase letters represent homology arms for homologous recombination; uppercase letters indicate barcode sequences used to replace the coding region.

| **Oligo code** | **Type** | **Sequence (5′ – 3′)** |
| --- | --- | --- |
| RIM15_sgRNA_FW | sgRNA | gactTTCAATAGAAGTAACACCGC |
| RIM15_sgRNA_RV | sgRNA | aaacGCGGTGTTACTTCTATTGAA |
| delRIM15_Donor_FW | Donor | gtgttgtttttcattgtaaaaataagttgttgttcgtatcacagcTAACTAGCTGAgaataaaaactttgcttgaaagcaaatctgagaataatcatggat |
| delRIM15_Donor_RV | Donor | atccatgattattctcagatttgctttcaagcaaagtttttattcTCAGCTAGTTAgctgtgatacgaacaacaacttatttttacaatgaaaaacaacac |
| delACE2_Donor_FW | Donor | aaagctacgttgcagggagactcaagcaacagttaaagtgctagaagaaaTAACTAGCTGAgcaaaactcgaaccgcaccctttcaaacgaaactgatgctctctgacgaa |
| delACE2_Donor_RV | Donor | ttcgtcagagagcatcagtttcgtttgaaagggtgcggttcgagttttgcTCAGCTAGTTAtttcttctagcactttaactgttgcttgagtctccctgcaacgtagcttt |
| truncACE2_Donor_FW | Donor | ataaaagcagggatgatcgatatgctgctggcacgttcacgcctagaacaTAGCTAACTGAgcaaaactcgaaccgcaccctttcaaacgaaactgatgctctctgacgaa |
| truncACE2_Donor_RV | Donor | ttcgtcagagagcatcagtttcgtttgaaagggtgcggttcgagttttgcTCAGTTAGCTAtgttctaggcgtgaacgtgccagcagcatatcgatcatccctgctttta |
| ACE2_sgRNA_FW | sgRNA | gactTACTGGTCAATGGTACCCGG |
| ACE2_sgRNA_RV | sgRNA | aaacCCGGGTACCATTGACCAGTA |
